# Recent Advances in Hydrogel-Based Phototherapy for Tumor Treatment

**DOI:** 10.3390/gels9040286

**Published:** 2023-04-01

**Authors:** Shuaiqi Gan, Yongzhi Wu, Xu Zhang, Zheng Zheng, Min Zhang, Li Long, Jinfeng Liao, Wenchuan Chen

**Affiliations:** 1 State Key Laboratory of Oral Diseases and National Clinical Research Center for Oral Diseases, Med-X Center for Materials, West China Hospital of Stomatology, Sichuan University, Chengdu 610041, China; 2 Department of Oral Prosthodontics, West China Hospital of Stomatology, Sichuan University, Chengdu 610041, China; 3 Jinjiang Out-Patient Section, West China Hospital of Stomatology, Sichuan University, Chengdu 610041, China

**Keywords:** hydrogel, photodynamic therapy, photothermal therapy, antitumor

## Abstract

Phototherapeutic agent-based phototherapies activated by light have proven to be safe modalities for the treatment of various malignant tumor indications. The two main modalities of phototherapies include photothermal therapy, which causes localized thermal damage to target lesions, and photodynamic therapy, which causes localized chemical damage by generated reactive oxygen species (ROS). Conventional phototherapies suffer a major shortcoming in their clinical application due to their phototoxicity, which primarily arises from the uncontrolled distribution of phototherapeutic agents in vivo. For successful antitumor phototherapy, it is essential to ensure the generation of heat or ROS specifically occurs at the tumor site. To minimize the reverse side effects of phototherapy while improving its therapeutic performance, extensive research has focused on developing hydrogel-based phototherapy for tumor treatment. The utilization of hydrogels as drug carriers allows for the sustained delivery of phototherapeutic agents to tumor sites, thereby limiting their adverse effects. Herein, we summarize the recent advancements in the design of hydrogels for antitumor phototherapy, offer a comprehensive overview of the latest advances in hydrogel-based phototherapy and its combination with other therapeutic modalities for tumor treatment, and discuss the current clinical status of hydrogel-based antitumor phototherapy.

## 1. Introduction

Malignant tumors have emerged as the foremost contributor to mortality rates worldwide, serving as a significant impediment to the progressive trend of increasing life expectancy [1]. They are projected to continue as the primary etiology of premature death, defined as death before the age of 70, on a global scale. Malignant tumor tissue exhibits distinctive features such as vascular malformation, low pH, hypoxia, and others. Concurrently, malignant tumor cells possess unique properties including unrestrained proliferation, facile metastasis, and drug resistance [2]. At present, the foremost approaches for malignant tumor therapy comprise surgery, chemotherapy, and radiotherapy. Although surgical procedures can be useful in the management of tumors, complete removal of all malignant tumor cells may not be feasible [3,4,5]. Chemotherapy and radiotherapy, while effective in eliminating tumor cells, can also result in collateral damage to normal tissue [3,4,5].

The use of phototherapy in tumor treatment has garnered significant attention due to its minimal invasiveness, high effectiveness, and low probability of drug resistance [6]. Phototherapy has been employed for tumor management for a considerable duration of time, dating back to the early 1960s, shortly after its initial investigation in the treatment of retinal detachment [7]. The achievement of effective tumor ablation in phototherapy necessitates the utilization of high-powered lasers, ranging in magnitude up to hundreds of watts. This poses challenges related to its safety and logistical feasibility. Thus, to circumvent these limitations, phototherapies that utilize externally administered phototherapeutic agents to augment the efficacy of light-based treatments have been devised. Based on the distinct properties of different techniques and phototherapeutic agents involved, phototherapy can be broadly categorized into two main types, namely photothermal therapy (PTT) and photodynamic therapy (PDT) [8].

PTT is a treatment modality that utilizes photothermal agents, encompassing both endogenous chromophores within human tissues and exogenous agents [7]. The exogenous photothermal agents can be classified into various types, including inorganic materials (e.g., gold nanoparticles and graphene oxide) and organic materials (e.g., indocyanine green (ICG) and polydopamine) [9,10]. These agents can facilitate photothermal conversion when exposed to light radiation of specific wavelengths, leading to the release of heat that elevates the temperature of the affected region, consequently inducing cell death [11].

In contrast to PTT, PDT relies on the production of reactive oxygen species (ROS) to mediate its cytotoxic effects [12]. The three primary mechanisms of ROS-mediated tumor tissue destruction are direct tumor cell killing, induction of vascular damage, and activation of an immune response against the tumor [13,14,15]. PDT necessitates three key elements, namely a photosensitizer, molecular oxygen, and light [16,17,18]. The majority of photosensitizers employed in clinical settings are derived from porphyrins, chlorines, or dyes, and are structurally based on the tetrapyrrole framework [19]. The photothermal agents and photosensitizers can absorb light in the visible range (wavelengths of 400–700 nm) or near-infrared (NIR) range (700–1350 nm), and commercially available lasers, such as alexandrite lasers (720–800 nm), dye lasers (390–1000 nm), and diode lasers (630–1100 nm) can excite these agents to generate heat or ROS [20]. The placement of light can be further controlled using interventional techniques such as optical fibers and endoscopy, which can minimize off-target toxicity to surrounding tissues and avoid invasive procedures such as laparotomy and thoracotomy [20].

Although phototherapy has achieved significant success, its extended clinical application is limited by the evident phototoxicity of traditional phototherapeutic methods [7,21,22,23,24]. The main cause of phototoxicity is the uncontrolled distribution of phototherapeutic agents, which can cause off-target effects in normal tissues, including the skin, blood vessels, and liver, when exposed to natural light, ultimately resulting in the damage of normal cells. Additionally, the uncontrolled distribution of phototherapeutic agents can lead to lower accumulation in tumor cells, limiting the efficacy of phototherapy. Secondly, from a technical standpoint, selectively irradiating tumor cells is a challenge due to the proximity of normal tissues. As a result, healthy cells near the tumor may also be damaged. To overcome these limitations, there is a need to develop a method for the controlled and sustained delivery of phototherapeutic agents directly to tumor sites to facilitate effective antitumor phototherapy. Therefore, advancements in drug delivery systems, such as liposomes, micelles, nanoparticles, microspheres, and hydrogels, have provided significant opportunities for improving antitumor phototherapy. Among these systems, hydrogels have gained prominence due to their high biocompatibility, and have emerged as the preferred options [25].

**Figure 1:**
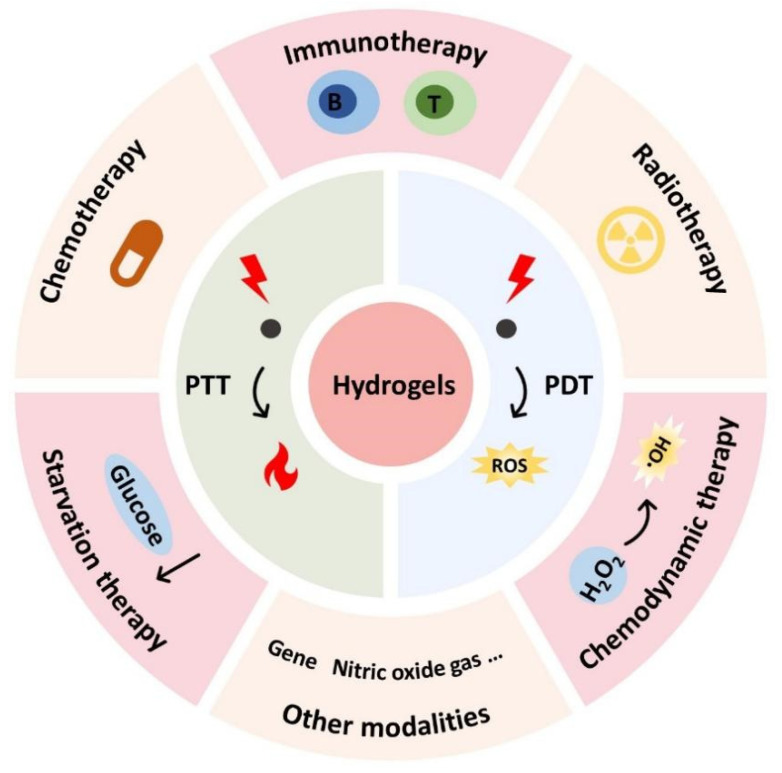
Schematic illustration of the hydrogels-based combinatorial phototherapy.

Hydrogels are insoluble biomaterial scaffolds with three-dimensional (3D) networks that can be formed through physical or chemical cross-linking [26,27]. Their unique features such as high biocompatibility, biodegradability, high water content, and flexible mechanical properties have made them extensively utilized in various biomedical applications, including tissue engineering, cell culture, drug delivery, biosensors, and antitumor therapy [28,29,30,31,32]. In recent years, hydrogels have garnered significant interest as drug carriers in antitumor phototherapy owing to their ability to achieve localized delivery of phototherapeutic agents while minimizing their distribution in non-target sites [33,34,35,36,37]. As a result, this approach can enhance therapeutic efficacy while reducing phototoxicity from phototherapy [38,39,40]. In addition, hydrogels also offer a versatile platform that shows significant potential to complement other tumor treatments such as immunotherapy, chemotherapy, and radiotherapy, and exhibit excellent synergistic antitumor efficacy (Figure 1) [41,42,43,44]. 

In this review, we will provide a summary of recent advancements in the design of hydrogels for antitumor phototherapy and offer a comprehensive overview of the latest advances in hydrogel-based phototherapy and its combination with other therapeutic modalities for tumor treatment. To illustrate these advances, representative examples will be presented. Lastly, the potential applications of hydrogels in clinical antitumor phototherapy will be discussed.

## 2. Hydrogels

Hydrogels are 3D networks of polymers that contain a significant amount of water or other fluids. Due to their high water content and cross-linked structure, hydrogels have potential for the encapsulation and delivery of photosensitizers/photothermal agents [41,42,43,44]. Furthermore, the characteristics of hydrogels, such as surface topography, porosity, and mechanical strength, depend largely on the properties of the polymers used and the mechanism of polymeric cross-linking [45,46]. By controlling various features of hydrogels, it is expected that a wide range of selectivities and diversities can be achieved for antitumor phototherapy. Hydrogels can be classified based on different parameters, including the source of materials (natural or synthetic), polymeric composition (homopolymeric, copolymeric, or multipolymeric), crosslinking mechanism (physical or chemical), sample size (nanogels, microgels, macrogels, or bulk hydrogels), and degradability (degradable or nondegradable) [47,48,49,50]. Considering the numerous excellent review articles on the introduction, classification, preparation, and application of hydrogels that have already been published [51,52,53,54,55,56,57,58], we will refrain from providing an exhaustive classification of hydrogels in this work.

For localized antitumor phototherapy, an injectable hydrogel is the preferred option for local administration. Compared to traditional hydrogels with pre-fabricated shapes, injectable hydrogels are better applied for filling irregular defects caused by tumor resection. They can also be injected into deep tissue through minimally invasive administration procedures [59,60,61]. Therefore, it is not surprising that most studies on hydrogel-based antitumor phototherapy are reported on the usage of injectable hydrogels for local hydrogel administration. Moreover, the use of conventional hydrogels in antitumor phototherapy is also limited by slow response rates, uncontrollable drug release, and difficulty in manipulating swelling and shrinking kinetics. To overcome these limitations, emerging hydrogels such as smart hydrogels and nanogels have garnered significant attention in antitumor phototherapy.

### 2.1. Smart Hydrogels

The distinctive characteristics of the tumor microenvironment, such as changes in overexpression of certain proteins and enzymes, acidic pH, hyperthermia, and redox potential have captured the attention of researchers who seek to utilize stimulus-responsive hydrogels, commonly known as smart hydrogels, for tumor treatment [62,63]. In contrast to conventional hydrogels, smart hydrogels have the unique ability to alter their physical or mechanical properties, such as swelling, shrinking, and undergoing a phase transition, in response to various stimuli, and thus make them suitable for various biomedical applications [64]. These stimuli can be external (such as temperature, magnetic fields, and light) or endogenous (such as enzymes, ionic strength, pH, and redox potential) [64,65,66,67,68,69,70,71]. This feature allows for on-demand drug release, leading to improved efficacy and reduced side effects from drug leakage [72,73,74,75,76]. Therefore, the utilization of smart hydrogels has become increasingly popular in the field of phototherapeutic agent-based phototherapies for tumor treatment [58]. In recent years, the widely used smart hydrogels for antitumor phototherapy are thermosensitive hydrogels, pH-sensitive hydrogels, photosensitive hydrogels, and redox-sensitive hydrogels.

1. Thermosensitive hydrogels. The application of thermosensitive hydrogels for antitumor phototherapy has undergone extensive research, with this type of smart hydrogel being the primary focus in the field. The unique sol–gel phase transition of thermosensitive hydrogels in response to temperature changes has led to their classification into two distinct groups: lower critical solution temperature (LCST) hydrogels and upper critical solution temperature (UCST) hydrogels [77,78]. The most commonly used thermosensitive hydrogels in recent years are LCST hydrogels, which are in a gel state above their LCST and a solution state below it. In the presence of temperatures lower than the LCST, the hydrophilic moieties present in the polymer chains of the hydrogel form hydrogen bonds with hydrophilic molecules present in their surroundings, causing the hydrogel to display high solubility. Upon temperature elevation, hydrogen bonds are weakened while the hydrophobic components of polymer chains experience increased interaction. This can lead to a substantial drop in polymer solubility, triggering matrix shrinkage or phase transition [79,80]. Furthermore, hydrogels with an LCST within the temperature range of room temperature to body temperature are highly suitable for in situ gelling upon injection. Additionally, the recent development of LCST hydrogels involves the incorporation of natural, such as chitosan [81], or synthetic macromolecules, such as poly-(N-isopropyl acrylamide) (PNIPAM) [82,83,84] and Pluronic F127 [85,86,87,88].
2. pH-sensitive hydrogels. Solid tumors are associated with an acidic tumor microenvironment, which is primarily due to the increased production of lactate by tumor cells resulting from their high rate of aerobic glycolysis [89]. The acidic nature of the tumor microenvironment has motivated the exploration of pH-sensitive drug delivery hydrogels to neutralize the microenvironment and impede tumor progression. Thus, pH-responsive hydrogels have been widely investigated as a localized drug delivery approach to achieve targeted antitumor phototherapy with reduced systematic toxicity. pH-responsive hydrogels can be divided into two main categories, namely anionic–cationic pH-responsive hydrogels and covalent pH-responsive hydrogels. The matrices of the anionic–cationic pH-responsive hydrogels typically comprise numerous weakly acidic or basic groups (e.g., carboxyl and amine groups) that can donate or accept protons. Alterations in the external pH can cause changes in the characteristics of these polymers, such as solubility and volume, which can lead to phase transitions and the release of drugs. In the case of in situ injection, the basic groups (such as amine groups) of the polymer undergo positive charge due to proton acceptance from the acidic external pH of the tumor microenvironment. Consequently, the polymer chains experience expansion and swelling from the electrostatic repulsion among charges. The enlarged mesh of the swollen hydrogels then facilitates the diffusion of drugs throughout the network. On the contrary, under acidic conditions, the presence of acidic groups (such as carboxyl group) in polymers causes the polymer chains to shrink. This shrinkage leads to the expulsion of water and drug molecules [90]. As for covalent pH-responsive hydrogels, the pH-sensitivity is attributed to degradation of acid-cleavable covalent bonds, such as ketals and hydra-zone bonds [91,92]. In addition, several natural polymers (such as chitosan, alginate, and cellulose) and synthetic polymers (such as polyamines and pyridine derivatives) have been extensively investigated for the production of pH-sensitive hydrogels [93]. Among these, chitosan and its derivatives are the most commonly employed polymers to develop pH-responsive hydrogels for antitumor phototherapy.
3. Photosensitive hydrogels. Photosensitive hydrogels can undergo property changes or a phase transition triggered by changes in their chemical structure or conformation under radiation, ultraviolet, or visible light [94,95]. Incorporating photothermal agents into hydrogel systems is a widely used method that involves photothermal conversion when exposed to light radiation, leading to a temperature increase in the system and a subsequent phase transition in thermosensitive hydrogels. This approach is also considered part of photothermal therapy. Another common method used to create light-responsive hydrogels involves polymers with a photoreactive moiety. This moiety can respond to light by undergoing photochemical reactions, such as photoisomerization, photocleavage, and crosslinking point photopolymerization. These reactions alter the properties of the hydrogel, including its crosslinking density, hydrophilicity, and charging state, which lead to phase transition and the release of drugs [93]. One example of a material used to prepare light-responsive hydrogels is gelatin methacryloyl (GelMA). This compound is created by incorporating methacryloyl groups into gelatin, which allows for photopolymerization by crosslinking with a photo-initiator [96]. Moreover, radiation can be used to trigger the swelling of hydrogels via ionizable functional groups. As a result of radiation-induced ionization, the osmotic pressure inside the hydrogel matrix increases, causing it to swell. This mechanism is similar to pH-sensitive hydrogels [95]. In recent years, the use of photosensitive hydrogels represents a promising approach for drug delivery in tumor treatment, as it enables noncontact drug delivery with high temporal and spatial precision. As a result, photosensitive hydrogels also have emerged as an attractive drug delivery system for antitumor phototherapy.
4. Redox-sensitive hydrogels. The current emphasis on redox-sensitive hydrogels is primarily centered on addressing tumor drug resistance and facilitating drug delivery. Redox-sensitive hydrogels can be designed to preferentially release drugs in the cytoplasm of tumor cells as opposed to the extracellular matrix and normal cells, primarily due to the higher levels of glutathione (GSH) in the former. Specifically, the cytoplasm contains a much higher concentration of GSH (2–10 mM) compared to the extracellular matrix (2–20 μM) [97]. In multidrug-resistant tumors, this difference is particularly pronounced, with the cytoplasmic GSH concentration being four times higher than in healthy tissues [98]. The incorporation of GSH-reactive groups (such as ditelluride, disulfide, and diselenide bonds) is a fundamental principle underlying such hydrogels, enabling their cleavage through the acceptance of electrons from the thiol groups in GSH [99,100].

### 2.2. Nanogels

Nanogels, which are colloidal particles of hydrogels at the nanoscale, have emerged as promising approaches for antitumor phototherapy [101]. Nanogels can be synthesized based on natural or synthetic polymers using a range of cross-linking techniques, including functional group cross-linking, radiation cross-linking, cross-linking polymerization, crystallization, and ionic cross-linking [102]. Furthermore, various nanogel configurations can be synthesized, such as yolk–shell architectures, double-walled structures, hollow nanogels, and core-shell nanoparticles [103,104,105,106,107]. Over the years, the biomedical applications of nanogels have been the subject of numerous review articles, many of which have examined the advancements made in drug delivery [108,109,110,111], regenerative medicine [112,113], imaging [114,115,116,117], diagnostics [118,119,120], theranostics [121,122], and tumor therapy [111,123,124,125].

It is worth noting that the ability of nanogels to cross biological barriers and deliver agents intracellularly through endocytosis makes them an attractive candidate for advanced drug delivery systems [126,127]. Nanogels also offer a solution to the problem of fast clearance of phototherapeutic agents in vivo by utilizing enhanced permeability and retention (EPR) effect to enhance their retention in tumors, thereby improving the therapeutic outcome of phototherapy for tumor treatment [7,21]. Specifically, the EPR effect is primarily attributed to the aggressive proliferation of tumor cells, which leads to the consumption of local nutrients and irregular blood vessel formation. In turn, the abnormal vessels contain porous openings that increase the ability of circulating nanogels to penetrate the tumor microenvironment. In contrast, non-malignant tissues are less penetrable due to the intact vasculature barrier. Furthermore, nanoparticles tend to accumulate selectively within tumor tissues due to the impaired lymphatic drainage system in those areas. In general, nanogels must have a size between 10 and 200 nm to achieve the EPR effect [128]. In addition to size, other intrinsic characteristics of nanogels, such as shape, electrical charge, hydrophilicity, and circulation time in the bloodstream, can impact the efficacy of the EPR effect [129]. While the effectiveness of EPR-based targeting has been demonstrated in preclinical tumor models in vivo, this approach has several drawbacks. These include limited efficacy for certain early-stage tumors due to their smaller size and more regular vasculature, heterogeneous throughout resulting from malformation of vessel fenestrations, and a lack of investigation of the EPR effect in human tumors [130,131].

Moreover, the large surface area of nanogels enables increased opportunities for multivalent bioconjugation and enhanced drug-loading capacity [132]. As a result of this unique nanostructure, nanogels demonstrate a rapid response to changes in the environment. Accordingly, smart nanogels, also called stimuli-responsive nanogels, can be designed to achieve triggered drug release in response to various stimuli, such as light, ion-exchange, pH, and temperature [133,134,135,136]. The use of smart nanogels as a drug delivery system for anti-tumor phototherapy holds great promise, as evidenced by recent reports on thermosensitive nanogels [137], pH-sensitive nanogels [138,139], photosensitive nanogels [140], and redox-sensitive hydrogels [139]. 

Ongoing research also indicates that smart nanogels could potentially revolutionize the field of tumor treatment by functioning not only as drug carriers but also as diagnostic, imaging, and theranostic agents [141]. Additionally, nanogels can be further customized to incorporate additional beneficial features, including prolonged circulation, targeted cell recognition, and multifunctionality through the integration of multiple features [126,142]. As a result, nanogels have emerged as one of the most promising platforms for phototherapeutic agent-based phototherapy.

## 3. Hydrogel-Based Phototherapy

### 3.1. Hydrogel-Based PTT

PTT involves the use of photothermal agents to generate localized heat [7]. Upon irradiation with light of a specific wavelength, the photothermal agents undergo photothermal conversion, which leads to an increase in kinetic energy and heating of the surrounding microenvironment. The extent of tissue damage induced by PTT depends on the temperature achieved. A heat-shock response is initiated at 41 °C [143], while irreversible tissue damage occurs at 42 °C [143,144]. Heating tissues to 42–46 °C for 10 min leads to cell necrosis, while cell death occurs almost instantaneously at tissue temperatures above 60 °C [145]. Although capable of eliminating established tumors, PTT may result in unintentional harm to healthy tissue adjacent to the tumor [146]. The incorporation of hydrogels in PTT can be beneficial in preserving the local temperature and reducing the risk of burns to healthy tissues. Furthermore, utilizing hydrogels to retain photothermal agents also enables repeated PTT sessions with a single administration of the hydrogel. 

**Figure 2:**
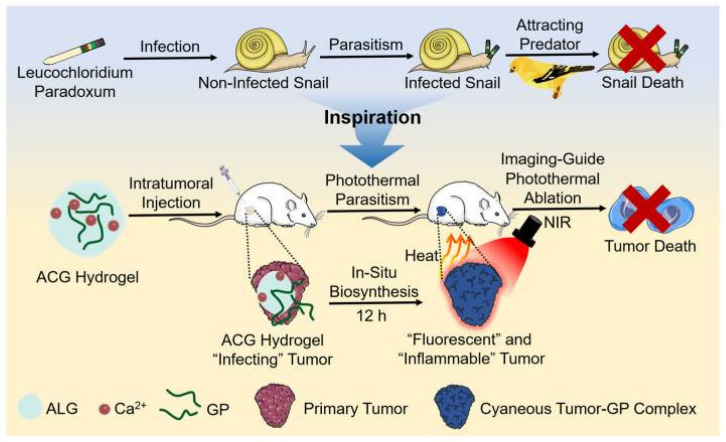
Schematic illustration of in situ biosynthesis of the genipin–protein crosslinking product for fluorescence imaging-guided PTT. Reprinted with permission from [148].

Hydrogels are widely used as carriers for investigating new photothermal agents for antitumor PTT. Yao et al. [85] achieved excellent antitumor PTT efficiency by incorporating titanium carbide nanoparticles as photothermal agents into thermosensitive Pluronic F127 hydrogels. Wang et al. [147] utilized an alginate-calcium hydrogel as a carrier to demonstrate the potential application of commercial copper sulfide as a photothermal agent for PTT. In another study, Wang et al. [148] prepared an alginate–calcium–genipin hydrogel that can achieve brilliant fluorescent and photothermal effects based on the fluorescent/photothermal features of the crosslinking product of genipin and protein (Figure 2). This hydrogel system provided a feasible solution for the homogenous dispersion of genipin and presented a novel methodological strategy for fluorescence imaging-guided antitumor phototherapy. However, it should be noted that the long-term biosafety of these photothermal agents should be carefully evaluated.

Self-assembled injectable hydrogels have received great attention in antitumor PTT. Hsiao et al. [149] modified polyaniline side chains into chitosan derivatives, which can self-assemble to form micelles and rapidly form hydrogels under the stimulation of a local acidic environment (pH = 6.9–7.0) in tumors. These hydrogels can then be loaded with hollow gold nanospheres as photothermal agents, which function as a heating source under NIR light irradiation. The local temperature of the tumor can quickly reach and be maintained at 50–55 °C within 5 min, surpassing the photothermal effect of commonly used inorganic photothermal agents, such as gold nanospheres. Additionally, the hydrogel system can prevent the leakage of the micelles and enable multiple effective treatments, thereby achieving the elimination of tumors and preventing tumor relapse. Moreover, Fan et al. [150] prepared an iodine-loaded starch-g-PNIPAM polymer that can self-assemble into nanogels and then transform into an injectable thermosensitive hydrogel at body temperature. The hydrogel showed an excellent photothermal effect due to the “iodine-starch” complex’s photothermal effect. Furthermore, the hydrogel can also exhibit bactericidal effects due to the release of iodine. However, the stability of these self-assembled hydrogels in the presence of body fluids should be further evaluated. Moreover, the intricate synthesis procedure involved in creating supramolecular hydrogels can impede their reproducibility and practical utilization. 

**Figure 3:**
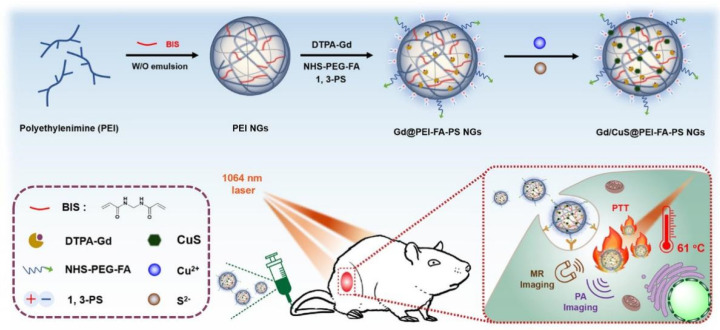
Schematic illustration of the synthesis of the PEI nanogel platform for tumor-targeted PTT. Reprinted with permission from [152]. Copyright 2023 American Chemical Society.

Nanogel-based PTT also has emerged as an attractive strategy for tumor treatment. Zhou et al. [151] reported the facile synthesis of a theranostic nanogel system for photoacoustic (PA) imaging-guided PTT of tumors. They used a double emulsion approach to synthesize γ-polyglutamic acid (γ-PGA) nanogels loaded with polyaniline (PANI). Then, they applied the carbodiimide coupling method to crosslink them with cystamine dihydrochloride (Cys). After loading with aniline monomers, the obtained γ-PGA/Cys nanogels underwent in situ polymerization to produce the final nanogel system. Additionally, this nanoplatform can be extended for diverse theranostic applications by functionalizing the surface carboxyl groups of the nanogels or by loading other theranostic elements. Furthermore, Zhang et al. [152] developed a polyethylenimine (PEI) nanogel system integrated with Gd and copper sulfide (CuS) for tumor-targeted PTT. The final cross-linked PEI nanogels were prepared using an inverse emulsion method. Under NIR light irradiation, the nanogel platform can realize magnetic resonance (MR) and PA imaging-guided antitumor PTT (Figure 3).

Hydrogels for tumor therapy are expected to degrade after achieving the therapeutic purpose. Therefore, hydrogels with an adjustable degradation rate have emerged as highly appealing options for antitumor therapy. Wang et al. [153] achieved efficient PTT on tumors by using alginate–calcium hydrogel as the carrier of the prepared photothermal agent, dendrimer-encapsulated platinum nanoparticles. Under NIR light irradiation, the hydrogel system can quickly heat up to 47 °C and maintain this temperature through multiple photothermal treatments, demonstrating a strong immobilizing effect of the hydrogel. After achieving the desired curative effect, the alginate–calcium hydrogel can be degraded by injecting chelates (DTPA), and the nanoparticles can be quickly cleared from the body through renal secretion. The adjustable degradation of the hydrogel system can greatly reduce the risk of toxicity from the long-term retention of the hydrogel in the body.

Following the completion of PTT, some localized hydrogel can also be employed to facilitate the tissue repair process. Liao et al. [154] incorporated gold nanorods and nanohydroxyapatite into a hydrogel composed of methacrylated gelatin/methacrylated chondroitin sulfate, resulting in a hydrogel system with remarkable PTT effects and bone-regenerative properties. Chen et al. [86] utilized a thermosensitive Pluronic F127 hydrogel as a carrier for carrageenan-capped gold–silver nanoparticles as a photothermal agent, aiming for the prevention of tumor recurrence and promotion of wound healing. However, the long-term safety of gold nanoparticle-encapsulated hydrogels in living animals is questionable due to the unknown cytotoxicity associated with gold nanoparticles. 

### 3.2. Hydrogel-Based PDT

PDT primarily induces tumor damage through the following mechanisms: (1) the direct killing effect of ROS on tumor cells, leading to apoptosis, necrosis, or autophagy; (2) photosensitizers target the tumor’s vascular system to form thrombi, resulting in hypoxic infarction of tumor tissues; and (3) the inflammatory response triggered by the release of inflammatory factors from apoptotic or necrotic tumor cells can promote an antitumor immune response [155,156,157,158]. The application of PDT necessitates the presence of three fundamental components: a photosensitizer, molecular oxygen, and light. Upon exposure to light, the photosensitizer absorbs photons, resulting in an excited electronic state. After excitation, the photosensitizer can transition to an excited singlet state and then undergo intersystem crossing to produce a long-lasting excited triplet state. The relaxation of the molecule can result in the emission of energy via fluorescence, heat, or other photophysical processes. Upon reaching the excited triplet state, the photosensitizer can induce the formation of ROS through two separate pathways. In the first pathway, the photosensitizer engages in electron transfer reactions to produce radicals and radical ions. In the second pathway, the photosensitizer transfers energy to triplet ground-state molecular oxygen (^3^O_2_), resulting in the production of highly reactive singlet oxygen (^1^O_2_) [159,160,161]. However, traditional PDT is limited by lack of targeting, aggregation in aqueous solutions, easy degradation, and instability of the photosensitizer, indicating the need for improvement to effectively kill tumor cells [162]. The incorporation of photosensitizers into hydrogels greatly enhances their localized concentration and biocompatibility, prolongs their residence time within the body, and ultimately augments the efficacy of PDT [163]. The incorporation of photosensitizers into hydrogels is a viable approach for enhancing the efficiency of photosensitizers [44,48].

The application of hydrogels as carriers has been extensively investigated for the evaluation of novel photosensitizers in the realm of antitumor PDT. Zhang et al. [164] incorporated TiO_2_ nanorods as a photosensitizer into PEGDA hydrogel. Under NIR light irradiation, TiO_2_ nanorods induced PEGDA molecules surrounding the tumor cells to wrap around the cell surface, while also generating a large amount of ROS to cause necrosis of the tumor cells. The presence of the hydrogel system also prevented TiO_2_ nanorods from flowing to normal tissues, reducing the possibility of side effects. In another study, Zhang et al. [81] synthesized a novel photosensitizer, NaYF_4_:Yb^3+^, Tm^3+^/Zn_2_GeO_4_:Mn^2+^/g-C_3_N_4_@hyaluronic acid (UZC@HA), and incorporated it into a thermosensitive chitosan hybrid hydrogel. UZC@HA exhibited tumor-targeting capability and could generate ROS upon irradiation with a 980 nm laser, leading to the destruction of tumor cells in the hydrogel. In a subcutaneous model of mouse breast cancer, intratumoral injection of the thermosensitive hydrogel demonstrated high antitumor efficacy with no side effects on normal tissues. However, the long-term toxicity of these novel photosensitizers should be thoroughly evaluated. 

**Figure 4:**
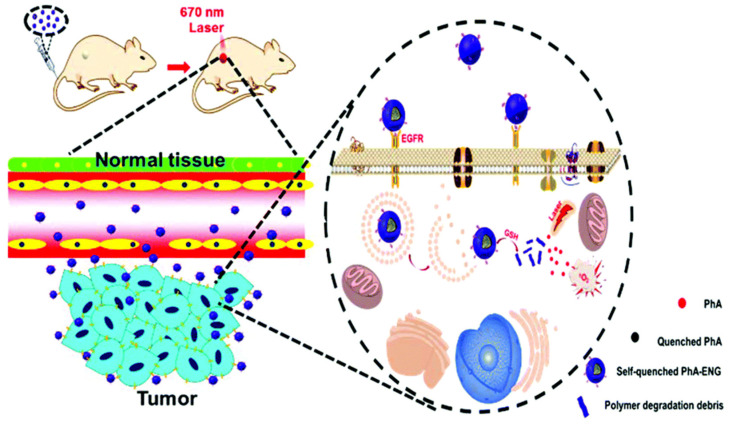
Schematic illustration of the smart self-quenched nanogel for targeted PDT. Reproduced from Ref. [166] with permission from the Royal Society of Chemistry.

Nanogels are also applied in PTT for efficient antitumor PDT. In the study conducted by Palantoken and colleagues [165], a nanoplatform for antitumor PDT was developed utilizing Ce6-bearing pullulan nanogels. The resultant nanogel system demonstrated enhanced water solubility and stability of Ce6. Notably, the nanogel exhibited significantly greater anti-tumor effects compared to the commonly used photosensitizer photofrin, with a 780-fold increase in efficacy observed. He et al. [166] developed a smart self-quenched nanogel for targeted PDT (Figure 4), which is crosslinked using poly[(2-(pyridin-2-yldisulfanyl) ethyl acrylate)-co-[poly(ethylene glycol)]] (PDA-PEG) polymers conjugated with photosensitizers pheophorbide A (PhA) via a disulfide bond. The nanogel inhibited the generation of ^1^O_2_ through self-aggregation-induced fluorescence quenching of PhA. It is also decorated with an anti-fluorescence resonance energy transfer affibody to improve its tumor-homing ability. The nanogel was quenched in the bloodstream, where the GSH concentration was low, reducing phototoxicity to normal tissues. Upon accumulation in tumor tissues, the nanogel can be activated by an elevated GSH concentration and realize efficient antitumor PDT. However, the long-term degradability and toxicity of nanogels should be further evaluated. 

Although PDT shows potential in antitumor treatment, it faces limitations in treating multidrug-resistant tumor, such as protective autophagy following sub-lethal PDT [167,168] and the short lifetime and limited diffusion distance of ^1^O_2_ [44,169,170], which is the primary form of ROS generated by PDT. Zhang et al. [138] designed a supramolecular nanogel system for the delivery of the photosensitizer tetraphenylporphinesulfonate (TPPS) to overcome multidrug resistance in tumor cells. The nanogel was prepared through the self-assembly of TPPS, organosilica nanodots, and methoxy-poly(ethylene glycol)_113_-block-poly(l-glutamic acid sodium salt)_200_. By exploiting the endo/lysosomal entrapment mechanism, the photosensitizer-loaded nanogels can evade drug efflux pumps and be internalized by drug-resistant tumor cells. The pH-sensitive nature of the nanogels allowed them to aggregate in the acidic endosomes/lysosomes, leading to their retention in cells. Furthermore, the nanogel-mediated damage to lysosomes after PDT inhibited protective autophagy, resulting in improved therapeutic performance. However, it should be noted that the long-term biosafety of the nanogel needs to be further investigated.

Moreover, current PDT approaches are suboptimal for treating large and deep-seated solid tumors due to poor light penetration depth [171]. To overcome this challenge, several chemical solutions for designing new-generation photosensitizers [172,173,174] and physical solutions for choosing alternative light sources [175,176,177,178,179,180] have been proposed and investigated to achieve more efficient PDT. Recently, Yang et al. [171] proposed a novel bacterial solution to address the poor light penetration depth of PDT. They used modified bioluminescent bacteria as an internal light source to activate the photosensitizer chlorin e6 (Ce6), enabling even illumination of whole tumors, and embedded it inside an alginate–calcium hydrogel. This hydrogel-based approach demonstrated superior therapeutic efficacy over conventional PDT.

**Figure 5:**
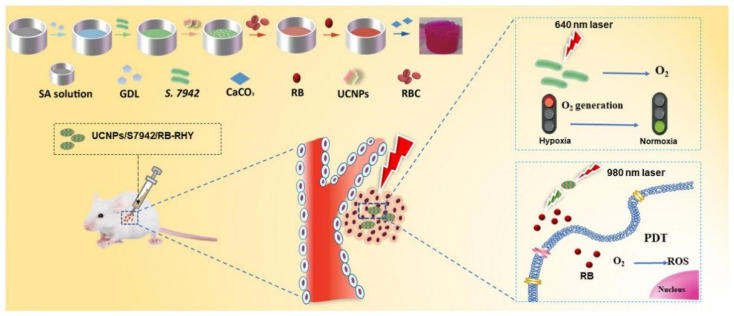
Schematic illustration of the preparation process of the novel anti-hypoxia hydrogel system for antitumor PDT. Reprinted with permission from [187].

Furthermore, another inherent problem of PDT is that its therapeutic effects depend on oxygen availability. Hypoxia is a typical feature of most tumor microenvironments caused by the rapid proliferation of tumor cells and abnormal growth of tumor blood vessels [181,182,183,184,185,186]. Currently, most photosensitizers consume a large amount of oxygen during PDT, which undoubtedly exacerbates tumor hypoxia and, in turn, significantly weakens the therapy’s effectiveness. The anti-hypoxia hydrogel system developed by Zhang et al. [187] presented innovative approaches to addressing the challenge of hypoxia in antitumor PDT. They used an injectable hydrogel made of red blood cell (RBC) membrane and sodium alginate to carry the photosensitizer Rose Bengal (RB), cyanobacteria Synechococcus elongatus PCC 7942 (S. 7942), and upconversion nanoparticles (UCNPs). In this anti-hypoxia hydrogel system, S. 7942 acted as a source of oxygen for PDT, while UCNPs facilitated the conversion of 980 nm light to visible light, triggering RB activation to release ROS for antitumor treatment (Figure 5). However, it is imperative to thoroughly assess the long-term biosafety of bacterial hydrogels. Moreover, the intricate process involved in synthesizing bacterial hydrogels could potentially hinder their reproducibility and widespread practical application.

### 3.3. Hydrogel-Based PDT Combined with PTT

The combination of PDT and PTT has been investigated as an efficient approach for enhancing the efficacy of antitumor phototherapy. By inducing heat, PTT can facilitate the intracellular transportation of photosensitizers and elevate the oxygen concentration in tumor tissue, which can result in enhanced efficacy of PDT [188,189,190,191]. Moreover, PDT-generated ROS can hamper the protective effects of heat shock protein (HSP) in tumor cells during PTT [192].

**Figure 6:**
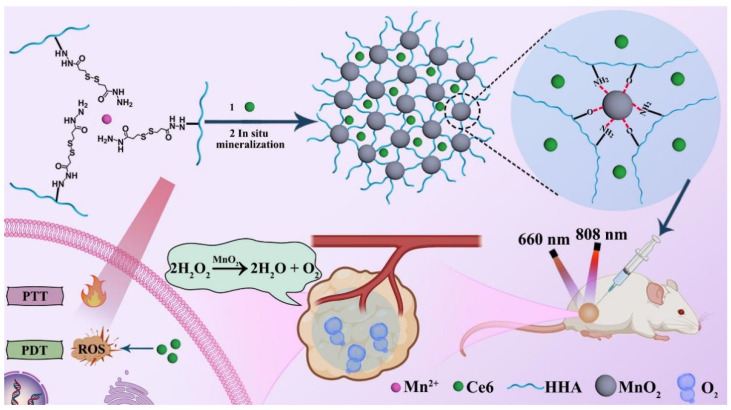
Schematic illustration of HHA-based hydrogel system for synergistic antitumor PTT/PDT. Reprinted with permission from [195].

Hydrogels are widely used as a platform for synergistic PTT/PDT of tumors. Yue et al. [193] prepared an injectable and self-healing hydrogel by combining Eu-doped red fluorescent carbon dots (CDs) with oxidized hyaluronic acid. The CDs were used not only as a cross-linking agent to form a hydrogel network through the Schiff base reaction but also as a photosensitizer/photothermal agent to realize efficient PTT/PDT synergistic treatment. This injectable hydrogel also showed good biocompatibility and provided a promising method for effective injectable hydrogel-based phototherapy for tumor treatment. Similarly, Qi et al. [194] synthesized an injectable and self-healing hydrogel through the formation of Schiff base bonds between hydroxypropyl chitosan and oxidized sodium alginate. After loading bovine serum albumin-modified molybdenum disulfide nanoflakes into the hydrogel system, it could realize excellent synergistic PTT/PDT of the tumor. In another study, Wang et al. [195] incorporated Mn^2+^ and hydrazided hyaluronan (HHA) to develop an injectable and self-healing hydrogel system. The hydrogel can be formed in a physiological pH condition through a mineralization-triggered Mn-hydrazide crosslinking. Incorporating photosensitizer chlorin e6 and manganese dioxide (MnO_2_) nanoparticles into the hydrogel enabled the accomplishment of oxygen-enhanced antitumor PTT/PDT synergistic treatment (Figure 6). However, the long-term toxic concerns of MnO_2_ nanoparticles should be thoroughly evaluated.

Additionally, Hou et al. [196] investigated the development of a novel thermosensitive agarose-based hydrogel, which was introduced with sodium humate, MnO_2_, and Ce6 to achieve effective PDT and PTT of tumors. The inherent characteristics of the thermosensitive hydrogels enabled their intra-tumoral injection, deep tissue penetration, and exceptional retention at the tumor site. Under NIR light irradiation, the photothermal agent sodium humate generated heat to kill tumor cells. The efficacy of PDT was enhanced by the produced heat, which facilitated the release of photosensitizer Ce6 and strengthened the catalytic effects of manganese dioxide on H_2_O_2_ to alleviate hypoxia. Furthermore, in vivo experiments have provided additional evidence regarding the ultralow systemic toxicity induced by the hybrid hydrogel.

## 4. Hydrogel-Based Phototherapy Combined with Other Therapeutic Modalities

The combination of phototherapy with conventional tumor therapies such as immunotherapy [197], chemotherapy [198], and radiotherapy [199] has shown significant potential for improving treatment outcomes, demonstrating strong synergistic antitumor effects. The natural imaging abilities of most organic photosensitizers and photothermal agents can provide phototherapy with diverse imaging functions, including PA imaging [200,201], MR imaging [202], and NIR fluorescent imaging [198]. The combination of diagnostic and therapeutic approaches is a promising way to improve the precision and efficacy of tumor therapy. Hydrogels possess a distinctive network structure and high water content, which enable them to carry both hydrophilic and hydrophobic molecules to achieve synergistic antitumor effects. Thus, hydrogel-based phototherapies can be used in conjunction with other antitumor therapies to improve their efficacy in treating tumors.

### 4.1. Hydrogel-Based Phototherapy Combined with Immunotherapy

The effectiveness of combining phototherapy and immunotherapy in tumor treatment has been extensively studied in recent years and also has been proven to be a highly synergistic approach [41]. Phototherapy can directly eradicate the tumor mass through photodynamic or photothermal effects and augment the effectiveness of immunotherapy. Evidence suggests that PDT can efficiently induce the release of danger-associated molecular patterns and tumor-associated antigens (TAAs) from tumor cells, which play a critical role in eliciting strong immune responses [203,204,205,206,207,208]. Additionally, PTT has been shown to promote immune responses and enhance the effectiveness of immunotherapy by triggering immunogenic cell death [209].

Hydrogels have recently emerged as a promising drug delivery system for the local administration of various immunotherapeutic agents in tumor immunotherapy [210]. They enable the targeted delivery of drugs to tumors, resulting in enhanced antitumor immunity at lower doses. Therefore, the codelivery of immunotherapeutic agents and phototherapeutic agents via hydrogels is anticipated to improve therapeutic efficacy by producing a combinational phototherapy-immunotherapy effect. For example, Dong et al. [211] developed a self-assembled injectable hydrogel system for combined antitumor photothermal/immunotherapy. The hydrogel was formed through the self-assembly of a copolymer of PEG and α-cyclodextrin, which was conjugated with New Indocyanine Green (IR820). Cytosine–phosphate–guanine (CpG) nanoparticles were fabricated separately and then added to the hydrogel system. Under NIR light irradiation, IR820 generated heat, which destroyed the tumor and induced antigens releasement from tumor cells. Additionally, CpG nanoparticles can regulate the immune function of various cells, including CD8^+^ T cells, dendritic cells, regulatory T cells, and myeloid-derived suppressor cells, resulting in a significant improvement in the effectiveness of the combined photothermal/immunotherapy treatment compared to single-modality therapy. Additionally, Fei et al. [212] developed an injectable hydrogel composed of RBCs for combined antitumor photothermal/immunotherapy. The hydrogel can be activated by physiological signals such as platelets and thrombin, and it has a deep-red color that enables it to act as a photosensitizer when heated by NIR light. Intratumoral injection of the hydrogel can achieve photoablation of tumors, producing debris of tumor cells and TAAs that have the potential to stimulate adaptive immune responses against tumor. The administration of the immune adjuvant imiquimod within the RBCs can elicit long-lasting and potent immune responses, which can prevent the spread and recurrence of tumor. Furthermore, recent studies also investigated the use of hydrogels, such as alginate–calcium hydrogel [213], alginate–collagen hydrogel [214], and gellan gum hydrogel [215], as carriers for phototherapeutic agents and immune stimulators in the context of combined antitumor photothermal/immunotherapy.

### 4.2. Hydrogel-Based Phototherapy Combined with Chemotherapy

Despite its widespread usage, chemotherapy, the most common tumor treatment, is faced with multiple challenges, including severe systemic side effects, inability to target tumor heterogeneity, low bioavailability, off-target effects, and drug resistance. PTT can generate heat that enhances the permeability of extracellular matrix, cell membranes, and blood vessels [216,217,218,219]. As a result, this physiological alteration increases the efficacy of chemotherapy by increasing the concentration of antitumor drugs within the tumor tissue [220]. Chemotherapy can also aid in improving the sensitivity of tumor cells to PTT in return [221,222]. In addition, the utilization of hydrogels as a drug delivery platform can enable efficient phototherapy as well as address the limitations of chemotherapy by enhancing the efficacy and reducing the toxicity of chemotherapy drug molecules [77,79,141]. 

The configuration of the hydrogel scaffold used for phototherapy can be designed using the 3D printing technique. Wei et al. [223] presented a simple and effective technique for producing core-shell hydrogel fibers/scaffolds that had potential application in PTT-chemotherapy for residual breast cancer treatment. The core-shell hydrogel fibers/scaffolds were created through a coaxial 3D printing technique, where concentrated alginate inks mixed with polydopamine as a photothermal agent were used for the shell layer, while the core consisted of temperature-sensitive gelatin hydrogels loaded with therapeutic drug doxorubicin (DOX). The NIR irradiation could cause a temperature increase, leading to a gel-sol transition of the core hydrogels and resulting in on-demand drug release. Moreover, using DOX, polydopamine nanoparticles, gellan gum, and sodium alginate, Xu et al. [224] created a 3D-printable hydrogel scaffold designed to achieve antitumor photothermal chemotherapy and promote wound healing following surgical intervention. 

**Figure 7:**
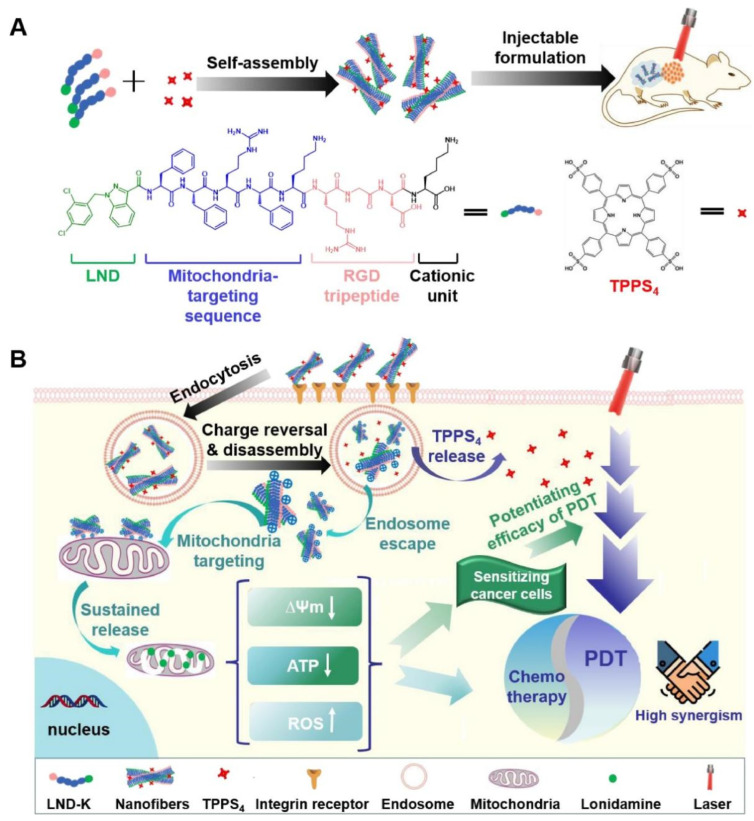
Schematic illustrations of self-assembled injectable hydrogels for combinational antitumor PDT-chemotherapy. (**A**) Injectable formulation of LND-K and TPPS_4_ for PDT. (**B**) Synergistic cancer therapy achieved by utilizing the self-assembled injectable hydrogel. Reprinted with permission from [226].

An injectable hydrogel is an ideal form of hydrogel for local hydrogel administration for phototherapy. The study conducted by Yang et al. [225] revealed that incorporating selenium as an anticancer agent and ICG as a photothermal agent into an injectable catecholamine-modified hyaluronic acid hydrogel can result in efficient PTT-chemotherapy for breast cancers. Additionally, Wu et al. [226] designed an injectable hydrogel composed of a lonidamine-conjugated peptide (LND-K) and photosensitizer TPPS_4_. The peptide self-assembled to form the hydrogel and enabled the targeted delivery of lonidamine to mitochondria for efficient photodynamic/chemotherapy combined therapy (Figure 7). In addition, Hou et al. [227] developed a nanogel system by combining an engineered polypeptide PC_10_A–arginine–glycine–aspartic acid (PC_10_ARGD) with Ag_2_S quantum dot as a photosensitizer. The nanogel system was subsequently loaded with DOX, a chemotherapy drug, and Bestatin, an immune-adjuvant drug, to transform into an injectable hydrogel. This strategy provides an effective combination of PTT and immunotherapy for the treatment of tumors. However, it should be noted that the long-term biosafety of Ag_2_S quantum dots needs to be further investigated.

**Figure 8:**
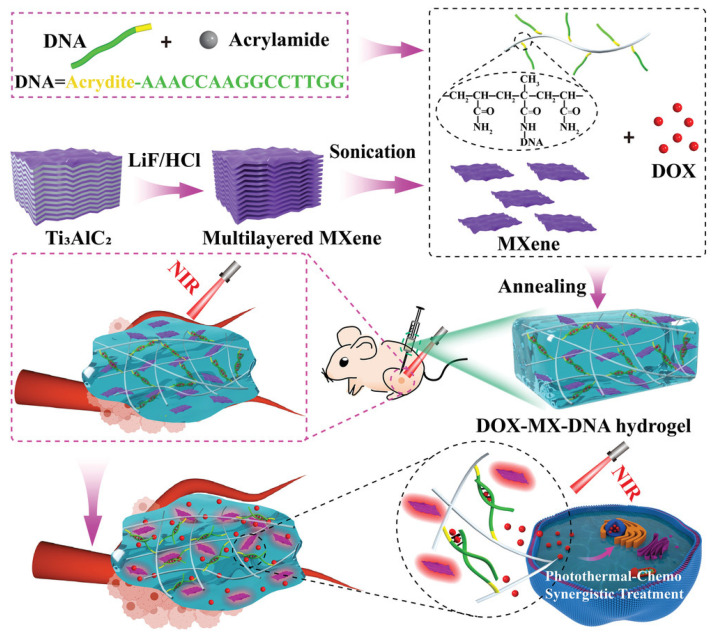
Schematic illustrations of self-assembled injectable hydrogels for combinational antitumor PDT-chemotherapy. Reprinted with permission from [229].

Thermosensitive hydrogels are the most popular smart hydrogels for synergistic PTT-chemotherapy. For hydrogels with good fluidity or thermosensitivity, PTT can regulate and control the release of chemotherapy drugs. For example, Huang et al. [87] developed a thermosensitive Pluronic F127 hydrogel platform incorporating self-assembled nanoparticles containing thermosensitive liposomes, gold–manganese oxide (Au–MnO) nanoparticles, and DOX. Under NIR light irradiation, the Au–MnO triggered the nanoparticles to release DOX into the hydrogel, while the sol–gel transition of the hydrogel at 37 °C avoided drug leakage and maintained sustained DOX release at the tumor site. Furthermore, Qi et al. [82] developed a thermosensitive hydrogel through the self-assembly of thermosensitive polymers PNIPAM, PEGDA, and 2,2-azobis [2-(2-imidazolin-2-yl) propane, which can undergo a sol–gel transition triggered by heat. After being loaded with ICG-methotrexate (MTX) nanomedicines, the hydrogel system exhibited additional desirable PTT efficiency and controlled release of MTX. Furthermore, Chang et al. [228] developed a thermosensitive hydrogel by combining thermosensitive polymers P(NIPAM-co-AH) and oxidized carboxymethyl cellulose. With the addition of a photothermal agent gold nanorods and DOX, the hydrogel system can realize the NIR-triggered photothermal effect and localized drug release of DOX. In another study, Zhang et al. [88] developed an injectable hydrogel system that incorporated chitosan polymeric micelles loaded with PTX and PEGylated gold nanorods in a thermosensitive Pluronic F127 hydrogel. This innovative approach enabled precise and selective thermal ablation of tumors while reducing the systemic toxicity of PTX. In addition, He et al. [229] synthesized a thermosensitive DNA hydrogel using acrydite-modified DNA and acrylamide monomers through conventional radical polymerization, which was integrated with DOX and a photothermal agent Ti_3_C_2_T_X_-based MXene nanosheets to establish the efficient antitumor PTT-chemotherapy (Figure 8). The gel-to-solution transition of the DOX-loaded MXene-DNA hydrogel can be triggered by a temperature rise resulting from the photothermal effect of MXene nanosheets upon NIR light irradiation. This process also involved the unwinding of DNA duplex crosslinking structures, enabling the on-demand release of DOX. Note that the long-term toxicity of Ti_3_C_2_T_X_-based MXene nanosheets should be thoroughly evaluated.

**Figure 9:**
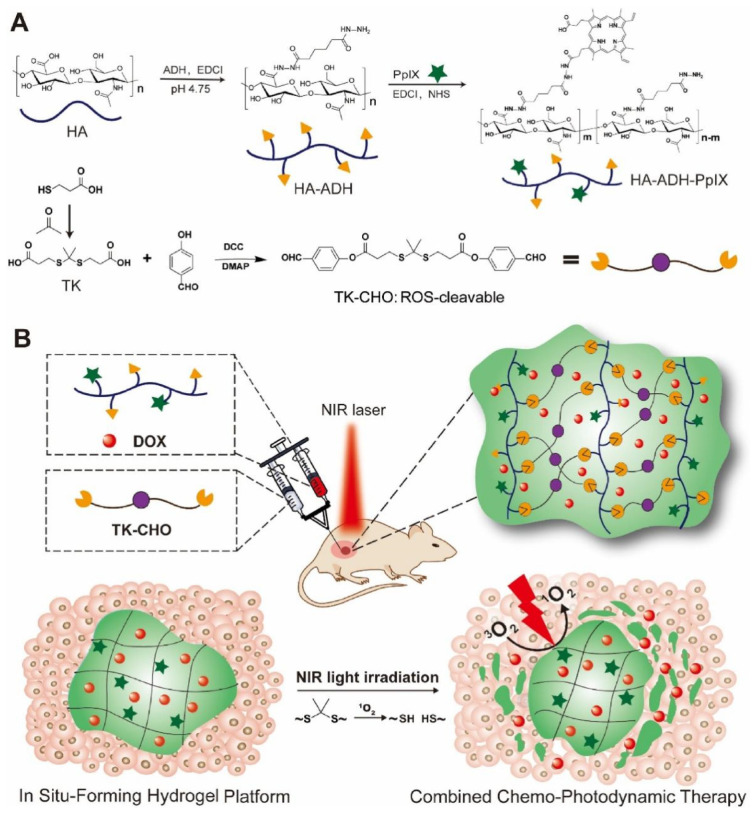
(**A**) Schematic illustrations for the synthesis of formulation of the ROS-sensitive hyaluronic acid hydrogel and (**B**) its application for combinational antitumor PDT-chemotherapy. Reprinted with permission from [231].

In addition to the aforementioned thermosensitive hydrogels, other types of stimulus-responsive hydrogels have also been investigated for combinational antitumor phototherapy-chemotherapy. Ghavaminejad et al. [83] incorporated DOX and photothermal agent dopamine nanoparticles, which were loaded with chemotherapy drugs bortezomib, into a thermosensitive pNIPAAm-co-pAAm hydrogel. Under the acidic tumor microenvironment, the boronic acid functionality of bortezomib would dissociate from the catechol groups of dopamine nanoparticles, thus leading to the release of this chemotherapy drug. As a result, the hydrogel can deliver drugs under an acidic environment as well as under NIR light irradiation. Zhang et al. [230] developed a silk sericin–chitosan hydrogel incorporating the chemotherapy drug tegafur (TF) and protoporphyrin IX (PpIX) heterodimers (TTP). The TTP formulation includes ROS-sensitive thioether bonds linking TF and the photosensitizer PpIX, allowing for gradual drug release upon the destruction of thioether bonds by high concentrations of ROS. The proposed approach achieved a synergistic effect between chemotherapy and PDT, while the on-demand drug release mechanism maximized the therapeutic effects of TF while minimizing its potential toxicity. Xu et al. [231] developed an injectable hyaluronic acid hydrogel that can be degraded in response to ROS and loaded it with PpIX as a photosensitizer and DOX as a chemotherapy drug. To achieve stable and efficient PDT, PpIX was covalently bonded to the hydrogel. Under NIR light irradiation, ROS generated by the photosensitizer can decompose the hydrogel, leading to an on-demand release of DOX (Figure 9).

**Figure 10:**
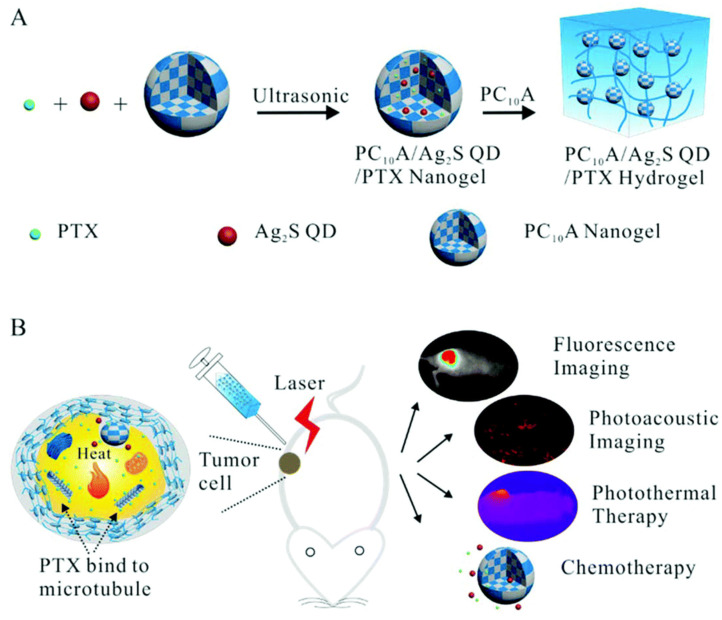
(**A**) Schematic illustration of the preparation of the injectable multifunctional PC_10_A hydrogel and (**B**) its application for combinational antitumor PTT-chemotherapy. Reproduced from Ref. [233] with permission from the Royal Society of Chemistry.

Nanogels as drug carriers also have been applied for combinational antitumor PDT-chemotherapy. The study by González-Ayón et al. [232] investigated the use of galacto-functionalized poly(N-vinylcaprolactam) (PNVCL)-based nanogels for encapsulating cisplatin or DOX and gold nanorods. The results demonstrated that the nanogels achieved an encapsulation efficiency of approximately 64% and 52% for cisplatin and DOX, respectively. This study highlighted the potential of PNVCL nanogels loaded with gold nanorods and cisplatin/DOX for enhancing PTT-chemotherapy. Jin et al. [233] developed an injectable multifunctional hydrogel as a theranostic platform for sustained antitumor PTT-chemotherapy. To achieve this, they prepared a nanogel system using an engineered polypeptide PC_10_A, which was loaded with oil-soluble PTX as a chemotherapy drug and Ag_2_S quantum dots (QDs) as photothermal agents. The PC_10_A hydrogel was then used to dissolve the nanogel system. The final hydrogel was found to be effective in inhibiting tumor cell growth, while NIR fluorescence and PA imaging enabled real-time monitoring of hydrogel degradation in vivo (Figure 10). However, the long-term biosafety of Ag_2_S QDs should be carefully evaluated.

Smart nanogels as promising carriers for combinational antitumor PTT-chemotherapy also have been widely investigated. Arjamaet et al. [139] synthesized hydrogel nanocubes composed of carboxymethyl-chitosan and PEG that were loaded with DOX-ICG, exhibiting pH and redox-responsive drug release behavior. The hydrogel nanocubes also integrated endosomal/lysosomal escape and rapid degradation in response to intracellular GSH into their design. Theune et al. [84] designed a nanogel system using thermosensitive polymers PNIPAM/PNIPMAM that incorporated the photothermal agent polymer polypyrrole (PPY) for combinational antitumor PTT-chemotherapy. The system was tested with MTX as a model antitumor drug and was found to be an efficient drug delivery system for chemotherapy. Zhou et al. [137] synthesized a DNA nanogel system by combining DOX as a chemotherapy drug, polyethyleneimine-black phosphorus quantum dots as photosensitizers/photothermal agents, and X-shaped DNA molecules through hydrogen and electrostatic bonding. This innovative approach allowed for the efficient on-demand delivery of DOX and combined PDT/PTT upon NIR light irradiation. Howaili et al. [140] synthesized a plasmonic nanogel with dual thermo-pH-responsiveness as a drug carrier system by grafting PNIPAM to chitosan using a chemical crosslinker. The incorporation of gold nanoparticles loaded with curcumin into the nanogel yielded a stimuli-responsive nanocarrier system with the potential for effective PTT-chemotherapy in tumor treatment.

### 4.3. Hydrogel-Based Phototherapy Combined with Radiotherapy

Radiotherapy is a widely utilized and effective tumor treatment technique that involves the use of ionizing radiation (e.g., γ-rays and X-rays) to destroy tumors by generating cytotoxic ROS [234]. The limited tissue penetration of light in phototherapy hampers its effectiveness in treating deep-seated tumors. In contrast, radiotherapy using high-energy X-rays can effectively penetrate the tissue and eliminate tumor cells located deep within the tumor tissue. However, the regulation of ionizing radiation dosage for eliminating tumors is challenging due to tumor cell radiation-resistance, which is related to the tumor hypoxic microenvironment, and the need to shield healthy tissues from the harmful effects of radiation exposure [235,236,237]. Fortunately, phototherapy can enhance the therapeutic outcomes of radiotherapy, overcoming some of its limitations [199,238,239]. The therapeutic efficacy of PDT and radiotherapy can be heightened when they are used in combination, and thus PDT can facilitate a reduction in radiation dose or exposure duration [240]. Additionally, PTT raises the temperature of the tumor tissue, which can enhance tumor oxygenation and heighten the sensitivity of the tumor to X-ray radiation [241].

Hydrogels are widely used as a platform for synergistic antitumor phototherapy/radiotherapy. For example, Mirrahimi et al. [242] incorporated cisplatin as a radiosensitizer and gold nanoparticles as photosensitizers and photothermal agents into an alginate hydrogel, resulting in successful and efficient antitumor thermo-chemo-radiotherapy. In another study, Wang et al. [243] investigated the use of agarose hydrogel containing Prussian blue nanoparticles for combined radiotherapy and PTT. The Prussian blue nanoparticles exhibited a high-efficiency photothermal effect under NIR light irradiation and could react with endogenous H_2_O_2_ to generate oxygen, thereby counteracting the hypoxic tumor environment and enhancing the sensitivity of tumor cells to radiotherapy. Furthermore, Zhou et al. [244] synthesized Cys-crosslinked γ-PGA nanogels and then incorporated photothermal agent PPY via oxidation polymerization under the influence of Fe(III) ions. They found that performing X-ray radiation after laser irradiation significantly increased the sensitization of the tumor to PTT. 

**Figure 11:**
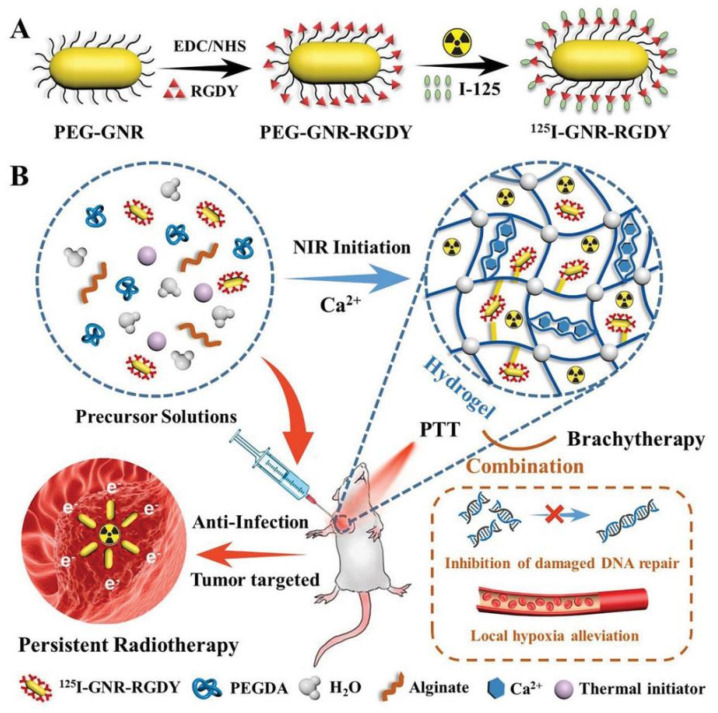
(**A**) Schematic illustration of the preparation of the modified gold nanorods and (**B**) the PEGDA-alginate double-network nanocomposite hydrogel and their application for combinational PTT-brachytherapy. Reprinted with permission from [247].

Brachytherapy is an important and useful type of radiotherapy that involves the implantation of small radioactive sources directly into or near the target tissue to destroy tumor cells [245]. Mukerji et al. [246] studied the use of photosensitizer Ce6 and radionuclide ^125^I in a hydrogel made from elastin-like polypeptides containing cysteine residues that can undergo ROS-mediated disulfide crosslinking. The hydrogel enabled stable fixation of ^125^I and improved safety in brachytherapy, while also showing a synergistic effect for PDT and brachytherapy. Moreover, Wu et al. [247] developed a PEGDA-alginate double-network nanocomposite hydrogel to provide a combinational PTT-brachytherapy therapy for preventing local recurrence and wound infection in postoperative breast cancer. They used ^125^I-labeled Arg–Gly–Asp–Tyr (RGDY)-modified gold nanorods as phototherapeutic agents to realize combinational PTT-brachytherapy effects. The double-network hydrogel was produced through a process that involved the initiation of PEGDA polymerization by heat and the formation of a second crosslink between alginate and Ca^2+^-alginate established in the tumor microenvironment (Figure 11). However, the development of nanocomposite hydrogels is a complex and multi-step process, and the long-term degradability and toxicity of the component should be thoroughly evaluated.

### 4.4. Hydrogel-Based Phototherapy Combined with Starvation Therapy

Starvation therapy is a therapeutic approach aimed at blocking the energy supply to tumor cells. It is a treatment approach that commonly utilizes glucose oxidase (GOx) or its mimicking enzymes to catalyze glucose into gluconic acid and H_2_O_2_, ultimately leading to glucose depletion and reduced ATP levels [248,249,250,251]. By decreasing ATP levels in tumor tissues, starvation therapy can downregulate HSP expression in tumor cells, which can enhance the efficacy of PTT [252,253,254]. Moreover, the H_2_O_2_ produced, which serves as a source of oxygen, can further improve the efficacy of PDT [255]. Despite its potential, GOx-mediated starvation therapy faces significant challenges such as safety concerns, immunogenicity, low stability, and fragile enzymatic activity, which limit its clinical applicability [256,257]. Thus, hydrogels have recently gained significant attention as a platform for combining phototherapy and starvation therapy.

**Figure 12:**
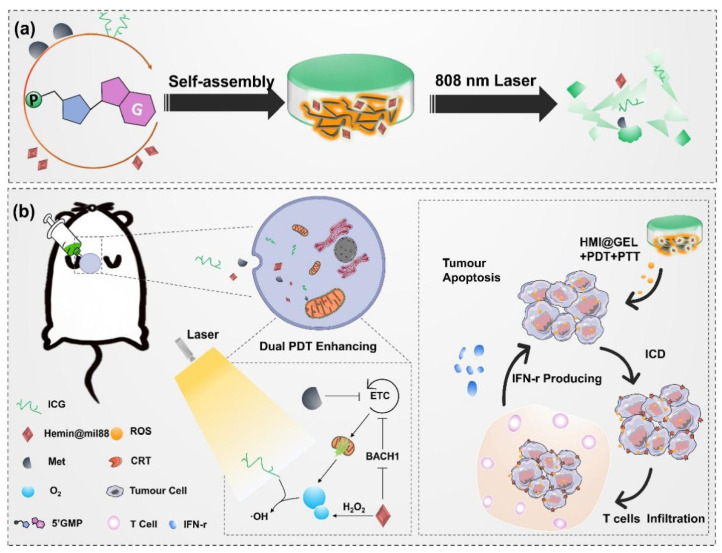
(**a**) Schematic illustration of the preparation of HMI@GEL hydrogel and (**b**) its application for combinational phototherapy-starvation therapy. Reprinted with permission from [259].

Recently, smart hydrogels have received much more preference. He et al. [258] developed a multifunctional hydrogel platform by incorporating GOx, a hydrogen peroxide catalytic active agent, and a photothermal agent prodrug into a thermosensitive PDLLA_1500_-PEG_1500_-PDLLA_1500_ hydrogel. This platform employed starvation therapy to block the energy supply of tumor tissue, thereby inhibiting the expression of HSP in tumor cells. By utilizing a relatively lower therapeutic temperature, the synergistic application of PTT and starvation therapy can achieve high tumor therapeutic efficacy while reducing the risk of burns to surrounding tissues and the local inflammatory response. Moreover, Sun et al. [259] fabricated a NIR light-responsive hydrogel by combining ICG as a phototherapeutic agent for PDT/PTT and 5′-guanosine monophosphate (5′GMP). Subsequently, they delivered a NIR light-responsive hydrogel system (HMI@GEL) after loading metformin (Met) to block the energy supply of the tumor and catalase-mimicking Hemin@mil88 to effectively alleviate tumor hypoxia. The hydrogel system achieved light-controlled local delivery of Met to the tumor site, which greatly reduced systemic side effects from Met leakage. In vivo antitumor experiments indicated that this hydrogel system could achieve excellent anti-tumor PDT/PTT efficacy (Figure 12).

**Figure 13:**
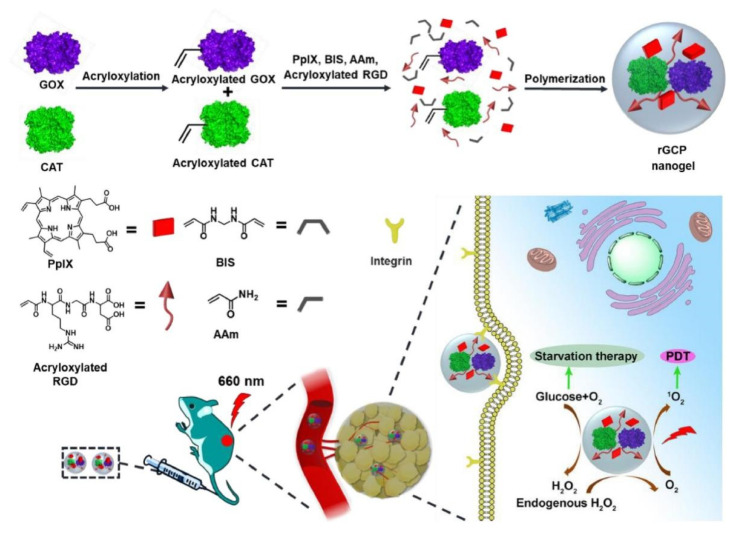
Schematic illustration of enzyme-immobilized nanogels for synergistic PDT and starvation therapy. Reprinted with permission from [261].

Nanogel-based platforms can also provide efficient combinational phototherapy-starvation therapy for tumor treatment. Luo et al. [260] and Fan et al. [261] created enzyme-immobilized nanogels with tumor tissue-targeting ability and self-supplying oxygen capability to counteract the rapid proliferation of cancer cells. The nanogel incorporating GOX and catalase (CAT) was fabricated via polymerization, utilizing PpIX and cancer-cell-specific Arg-Gly-Asp (RGD) as comonomers (Figure 13). Inside the nanogel, the cascade reaction efficiently utilized intracellular glucose catalyzed by GOX, while CAT safely decomposed the generated H_2_O_2_ to generate oxygen. The production of oxygen could further enhance glucose consumption by GOX and facilitate the generation of ^1^O_2_. Combining starvation therapy and PDT, the nanogel system exhibited a potent synergistic effect against cancer cells. However, the stability and degradability of the nanogel should be further evaluated.

### 4.5. Hydrogel-Based Phototherapy Combined with Chemodynamic Therapy

Chemodynamic therapy is a promising tumor treatment modality that generates cytotoxic hydroxyl radicals via the catalysis of metal ions on H_2_O_2_ through a Fenton or Fenton-like reaction, and unlike oxygen-dependent PDT, it does not rely on the availability of oxygen [262,263,264,265]. For example, Chen et al. [266] incorporated Hu Kaiwen ink as a photothermal agent and dihydroartemisinin-Fe^2+^ as an inducer of ROS into an agarose hydrogel to achieve synergistic antitumor PTT-chemodynamic therapy. Moreover, Qin et al. [267] prepared an enzyme complex-loaded hybrid nanogel that could function in diverse biomedical applications related to ROS regulation and could serve as a safe and effective PDT-chemodynamic therapy for tumors.

**Figure 14:**
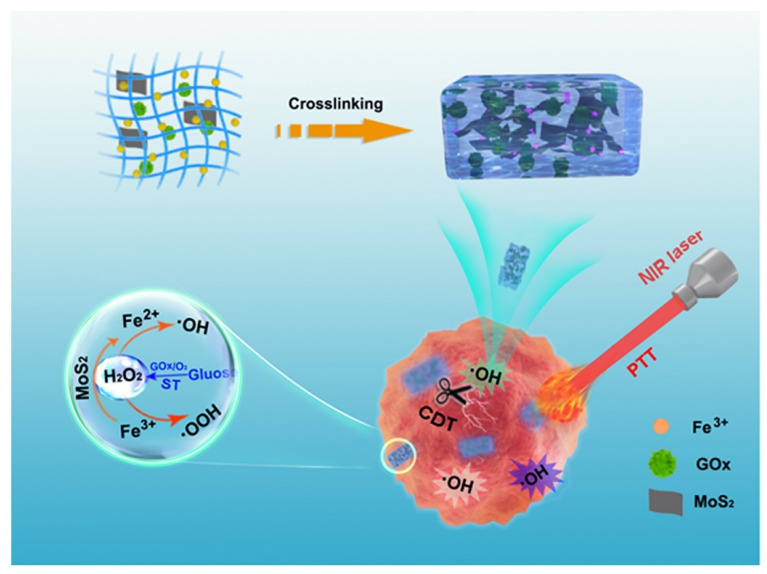
Schematic illustration of the sodium-alginate hydrogel system for combinational photothermal/starvation/chemodynamic therapy. Reprinted with permission from [273].

However, chemodynamic therapy alone is often insufficient to achieve a satisfactory antitumor effect due to the limited H_2_O_2_ in the tumor microenvironment [268,269,270,271,272]. Therefore, it is often combined with GOx-mediated starvation therapy, which can produce H_2_O_2_, to achieve the desired therapeutic outcome. For example, Zhou et al. [273] wisely designed a novel sodium-alginate hydrogel system for combined cancer photothermal, starvation, and chemodynamic therapy. This hydrogel encapsulates molybdenum dioxide (MoS_2_) nanosheets as a photothermal agent, GOx that mediates starvation therapy, and Fe^3+^ as a cross-linking agent. In this hydrogel system, MoS_2_ nanosheets had a strong photothermal ability and can convert Fe^3+^ to Fe^2+^. GOx can catalyze glucose to produce gluconic acid and H_2_O_2_, and the Fe^2+^ can undergo a Fenton reaction with excess H_2_O_2_ to produce hydroxyl radicals, achieving an enhanced therapeutic effect (Figure 14). In another study, Xu et al. [274] utilized a gelatin-hydroxyphenyl hydrogel as a carrier for the enzyme CoMnFe-layered double oxides (CoMnFe-LDO) and the GOx. Due to the cascaded catalytic reaction performed by CoMnFe-LDO and GOx, the composite hydrogel can realize satisfactory synergetic multifunctional tumor therapies, including chemodynamic therapy, starvation therapy, and PTT. Furthermore, Lee et al. [275] developed a hyaluronic acid-based hydrogel for the treatment of breast cancer through multiple approaches including ferroptosis, PTT, chemodynamic therapy, and starvation therapy.

### 4.6. Hydrogel-Based Phototherapy Combined with Other Antitumor Modalities

In addition to the above-mentioned combinational strategies for tumor treatment, researchers have also explored antitumor modalities that combine phototherapy with other antitumor treatments, such as gene therapy, nitric oxide (NO) gas therapy, thermodynamic therapy, anti-angiogenic therapy, and epigenetic therapy.

Conde et al. [276] simultaneously loaded small interfering RNA, gold nanorods, and Avastin into a dendrimer/dextran hydrogel for a synergistic gene/drug/phototherapy. Their study demonstrated that modified gold nanorods as phototherapeutic agents, which were used as small interfering RNA carriers, could significantly knock down cancer-promoting genes. Additionally, modified gold nanorods were also used to load antitumor drugs Avastin and enhance targeting drug delivery. This three-mode synergistic treatment strategy of phototherapy-chemotherapy-gene therapy has the potential to eliminate tumors and prevent a recurrence, making it a promising candidate for clinical applications.

Sun et al. [277] developed an injectable and thermosensitive hydrogel system that enabled combined PDT-NO gas therapy. The hydrogel system was based on a poly(ε-caprolactone)-poly(ethylene glycol)-poly(ε-caprolactone) (PCL–PEG–PCL) hydrogel, which served as a carrier for poly(lactic-glycolic acid) (PLGA) nanoparticles encapsulating both the photosensitizer ICG and NO donor l-arginine. The system induced ROS production and promoted NO production, leading to cancer cell apoptosis and inhibition of cancer cell proliferation. However, the long-term biosafety of PCL–PEG–PCL copolymer should be thoroughly evaluated, and the large-scale production of this hydrogel system seemed to be challenging.

To combine PTT-thermodynamic therapy for hypoxic tumor treatment, Sun et al. [278] developed an innovative injectable hydrogel formed by copolymerizing hydrophilic glycidyl methacrylate-modified hyaluronic acid with hydrophobic N-isopropyl acrylamide. The addition of 2,2′-Azobis [2-(2-imidazalin-2-yl)propane] dihydrochloride (AIPH) as an alkyl-free radical source and gold nanorods as photothermal agents enabled the hydrogel to generate free radicals from AIPH and heat through the photothermal effects, providing an efficient strategy for treating hypoxic tumors.

To realize combined PTT and targeted anti-angiogenic therapy for tumor treatment, Wu et al. [279] designed an injectable gellan gum hydrogel that integrated ultra-small bismuth sulfide (Bi_2_S_3_) nanodots as photothermal agents and sorafenib as a targeted therapy drug. However, the long-term safety of Bi_2_S_3_ nanodots-encapsulated hydrogels is questionable due to the unknown cytotoxicity associated with Bi_2_S_3_ nanodots.

To achieve synergistic PDT/epigenetic therapy for prostate cancer, Liu et al. [280] devised a smart therapeutic nanoplatform that used a nanogel as a photosensitizer and drug carrier. By suppressing HIF-1α and VEGF pathways that contribute to PDT resistance, the inclusion of histone deacetylase inhibitors into PDT enhanced the synergistic PDT/epigenetic therapy for prostate cancer. The study highlighted the well-designed architecture of the nanoplatform, which functioned as a photodynamic agent without releasing Ce6 photosensitizer molecules in a responsive environment. It also allowed for the incorporation of diverse functional components for smart drug release and imaging-guided combination therapy.

## 5. Conclusions and Prospect

In recent years, significant progress has been made in the development of hydrogels for antitumor phototherapy, which shows great potential for clinical applications in tumor treatment. The use of hydrogels in antitumor phototherapy offers unique advantages, including (1) the ability to encapsulate phototherapeutic agents to enhance their therapeutic efficacy and reduce their side effects; (2) easy access to the target site via needle injection with injectable hydrogels; (3) the ability of smart hydrogels to release phototherapeutic agents on-demand; (4) the selective accumulation of nanogels in tumors through EPR effect; (5) the capability of nanogels to deliver phototherapeutic agents intracellularly, with potential tumor-targeting and multifunctionality features; and (6) the capacity of hydrogels as a carrier to synergistically combine with other therapeutic modalities to achieve a more effective therapeutic effect.

However, hydrogel-based phototherapies face some limitations in clinical tumor treatment. First, the in vivo stability and degradability of phototherapeutic hydrogel systems remain uncertain and concerning. The impact of prolonged exposure of hydrogels on tumor sites and the mechanism of its phototherapeutic effect are not yet to be fully understood. Additionally, there is a lack of clarity regarding the long-term toxic and side effects of phototherapeutic agents and hydrogels on organisms in current studies. Second, the therapeutic efficacy of most hydrogels-based phototherapies lacks rigorous assessment or verification in tumor models. Moreover, the commonly used mouse tumor models in preclinical experiments are not adequate to reflect the physiological conditions of clinical patients. To advance the clinical translation of hydrogels-based phototherapies, it is necessary to assess their therapeutic efficacy in larger animals, such as dogs, pigs, and monkeys.

Furthermore, the design, preparation, and application of phototherapeutic hydrogel systems are still in the experimental stage due to the current lack of clinical investigations. To date, no hydrogel-based antitumor phototherapy has been registered on the Clinical Trials Gov database (http://www.clinicaltrials.gov (accessed on 26 March 2023)). Furthermore, compared with bulk hydrogels, the clinical application of nanogels is a relatively new area of research, with only one nanogel-related therapeutic modality entered clinical trials (ClinicalTrials.gov Identifier: NCT05268718). Many therapeutic factors (such as questionable biosafety and efficacy) and non-therapeutic factors (such as lack of clear regulatory guidelines and standardized assays) hinder the clinical transition of nanogels [126]. Therefore, compared with nanogels, bulk hydrogels-based phototherapies are more promising to enter the clinical stage first.

In conclusion, hydrogels are promising carriers for antitumor phototherapies, but further in-depth research is required to establish the safety and effectiveness of hydrogel-based phototherapy for tumor clinical treatment. Although the current understanding is limited, there is a growing expectation that hydrogel-based phototherapy will soon experience a significant surge in clinical applications. Hence, it is crucial to continuously explore the possibilities offered by hydrogel-based phototherapy to optimize its future clinical applications in tumor treatment.

## Data Availability

Not applicable.
